# Examining the impact of schizotypal personality traits on event-related potential (ERP) indexes of sensory gating in a healthy population

**DOI:** 10.1017/pen.2023.1

**Published:** 2023-04-27

**Authors:** Ashley M. Francis, T-Jay Anderson, Lauren Ross, Jenna N. Bissonnette, Kaitlyn R. Napier, N. W. Shead, Derek J. Fisher

**Affiliations:** 1 Department of Psychiatry, Dalhousie University, Halifax, Canada; 2 Department of Psychology and Neuroscience, Dalhousie University, Halifax, Canada; 3 Department of Psychology, Mount Saint Vincent University, Halifax, Canada

**Keywords:** Sensory Gating, Attention allocation, Schizotypal Traits

## Abstract

The aim of this study was to better understand the relation of schizotypy traits with sensory gating ability in a sample of community-dwelling individuals with high and low schizotypy traits. Sensory gating was assessed through the paired click paradigm and mid-latency evoked responses (i.e., P50, N100, P200), while schizotypy traits were assessed through the SPQ-BR which was used to classify participants into “high” and “low” schizotypy groups. Based on prior work, we hypothesized that those with the highest schizotypy scores would have reduced sensory gating ability. While this study does not show differences between relatively low and high schizotypy groups on sensory gating ability, it does suggest that our participants may have been experiencing deficits in attention allocation, a downstream cognitive processing measure. Scores on the SPQ-BR suggest that our sample was not close to the high end of the schizotypy traits which may help explain why no differences were found. This research shows the importance of including all levels of schizotypy ratings in clinical research as we can gain a clearer view of the impact of schizotypy on the brain and cognitive functioning in those with “high” levels of schizotypy. Additionally, this work highlights the importance of including measures of important factors such as impulsivity and sensation-seeking to better understand what aspects of schizotypy may be driving these sensory gating alterations reported in the literature.

## 1. Introduction

Individual differences in personality traits are an important area in electrophysiological research as they predict differing brain activity (Fajkowska, Eysenck, Zagórska, & Jaśkowski, 2011). One such differentiating cluster of personality traits is schizotypy, which closely resembles symptoms of schizophrenia (Claridge & Beech, 1995). Individuals with high schizotypy traits display increased difficulties with social functioning (Aguirre et al., 2008; Asher et al., 2013; Jahshan & Sergi, 2007; Pedrero & Debbané, 2017), sensory gating, impulse control as well as other deficits in cognitive functioning (Evans, Gray, & Snowden 2007; Sumich et al., 2008; Wan, Crawford, & Boutros 2006). These alterations in cognitive functioning and personality traits seen in a healthy population may indicate a predisposition for schizophrenia or related spectrum disorders (Kwapil & Barrantes-Vidal, 2015; Wan et al., 2017). Prior work has suggested that while schizotypy traits vary in severity in a healthy population, approximately 5% of the general population are estimated to have high schizotypy and subclinical levels of psychosis (Rössler et al., 2015; Van Os et al., 2009).

The cognitive deficits found in healthy controls with high schizotypy traits resemble the same alterations found in those with schizotypal personality disorder, schizophrenia, and those at high risk for schizophrenia (e.g., genetic or clinical high risk; Clementz, Geyer, & Braff 1998; Croft, Lee, Bertolot, & Gruzelier, 2001; Croft, Dimoska, Gonsalvez, & Clarke 2004; Myles-Worsley, Ord, Blailes, Ngiralmau, & Freedman 2004; Olincy et al., 2010; Wan, Crawford, & Boutros 2007, Wan et al., 2006). Specifically, individuals who score high in self-reported characteristics of schizotypy present with similar, yet less severe psychological and biological abnormalities when compared to individuals with schizophrenia (Mohanty et al., 2005). As such, the study of schizotypy allows us to better understand how related traits impact brain function along a continuum and without the potentially confounding effects of medication.

Key features of schizotypy traits include deficits in attention, information processing and sensory gating, or the inability to gate out irrelevant sensory information (i.e., sensory gating; Clementz et al., 1998; Croft et al., 2001, 2004; Hazlett et al., 2015; Myles-Worsley et al., 2004; Olincy et al., 2010; Wan et al., 2007, 2006). Recent research has shown that different personality variants can also be predictive of one’s efficiency in switching attention (De Pascalis & Speranza, 2000), specifically sensation-seeking behaviors and high levels of impulsivity have been linked to sensory gating deficits (Houston & Stanford, 2001; Lawson et al., 2012; Lijffijt et al., 2012; Zheng et al., 2010). Both sensation-seeking and high levels of impulsivity are also found in those with increased schizotypy traits (Del Giudice et al., 2014); thus, sensory gating deficits would be expected in individuals with high levels of schizotypy, as well as those with an increase in sensation-seeking behaviors and impulsivity.

Sensory gating allows for normal information processing and the avoidance of sensory overload (Adler et al., 1998). A failure to inhibit irrelevant sensory stimuli can result in higher-order processing being overwhelmed by irrelevant stimuli, which has been associated with schizophrenia spectrum disorders (Braff & Geyer, 1990; Clementz et al., 1998; McGhie & Chapman, 1961; Olincy et al., 2010). Some research even suggests that these deficits in sensory gating ability are associated with the behavioral symptoms observed in those with schizophrenia (i.e., sensory overload and psychotic symptoms; Waters, Badcock, Maybery, & Michie, 2003).

One of the more common methods typically used to measure sensory gating and the associated brain networks is the auditory paired click paradigm (Boutros & Belger, 1999). The paradigm uses a series of clicks presented within 500 ms of each other, the first click (S_1_) is known as the conditioning stimulus and activates the inhibitory circuits in the brain, while the second click (S_2_) is the “test” click and tests the strength of this inhibitory brain mechanism (Eccles, 1969). While there are several electroencephalography (EEG)-derived event-related potentials (ERPs) that the paired click paradigm elicits, the most common is the P50. The P50 is a positive ERP that reaches peak amplitude around 50 ms post-stimulus presentation and is an index of pre-attentive processing that requires no conscious awareness. The paired click paradigm also elicits the N100 and P200, a series of negative (N100) and positive (P200) going waveforms that reach maximal amplitude at 100 and 200 ms post-stimulus presentation. The N100 is an index of later stream attention filtering while the P200 is an index of attention allocation; these three ERPs together make up the mid-latency auditory evoked responses (MLAERs).

Given the increasing demand to better understand and support individual differences in both academic and work environments, the main goal of this study was to investigate how traits of schizotypy, impulsivity, and sensation-seeking are associated with brain functioning, specifically sensory gating ability and attention allocation and filtering using EEG. Based on prior work (Clementz et al., 1998; Croft et al., 2001, 2004; Hazlett et al., 2015; Myles-Worsley et al., 2004; Olincy et al., 2010; Wan et al., 2006, 2007), we anticipated that those with high schizotypy traits would have worse sensory gating and attentional ability, as indexed by the P50, N100, and P200 ERPs, relative to those with low schizotypy traits. This will ultimately help to better identify the relative strengths and weaknesses of individuals with high and low levels of these traits.

## 2. Methods

### 2.1. Study participants

All participants (*N* = 31) were between the ages of 18 and 42 years, were fluent in English, right-handed, and had normal or corrected-to-normal vision and hearing. All participants were recruited within Nova Scotia through a community sample and word-of-mouth with most of our participants being gathered from a predominantly university student sample. A summary of participant demographic data can be found in Table 1.

**Table 1.** Demographic information for low and high schizotypy groups

	Low Schizotypy M (SD)	High Schizotypy M (SD)	*t*-value (df)	*p*- value (*g*-value)
Gender				
Male(Female)	7 (9)	6 (9)	χ^2^(1,N=31) = .045	*p* = .56
Age	25.31 (4.70)	24.87 (6.64)		
SPQ	41.43 (9.73)	69.20 (10.84)	−7.51 (29)	*p* < .001
CP	18.88 (4.96)	31.33 (3.75)	−7.84 (29)	*p* < .001
IP	7.63 (2.58)	12.27 (2.40)	−5.17 (29)	*p* < .001
DO	10.25 (3.45)	17.47 (5.46)	−4.43 (29)	*p* < .001
SA	4.69 (2.30)	8.13 (2.53)	−3.97 (29)	*p* < .001
Sensation Seeking Scale (total)	19.67 (6.64)	19.19 (6.74)	0.19 (29)	*p* = .85
SSS Dis	5.53 (2.38)	4.53 (1.64)	1.34 (29)	*p* = .19
SSS Bor	2.13 (1.85)	2.13 (2.29)	0.00 (29)	*p* = 1.0
SSS Thril	4.53 (3.16)	4.53 (3.31)	0.00 (29)	*p* = 1.0
SSS Experience	5.47 (1.92)	6.00 (2.17)	−0.71 (29)	*p* = .48
BIM - Attention	10.19 (2.61)	10.53 (3.20)	−0.33 (29)	*p* = .74
BIM – Cognitive Instability	6.81 (1.68)	6.73 (1.33)	0.15 (29)	*p* = .89
BIM - Motor	12.81 (2.56)	16.29 (3.00)	−3.42 (29)	*p* = .002
BIM – Perseverance	6.94 (1.77)	7.13 (1.68)	−0.32 (29)	*p* = .76
BIM – Self Control	10.19 (2.59)	11.60 (3.29)	−1.33 (29)	*p* = .19
BIM – Cognitive Complexity	9.63 (2.25)	11.07 (2.25)	−1.78 (29)	*p* = .085
BIM – 2^nd^ order Attentional	17.00 (3.16)	17.27 (3.33)	−0.23 (29)	*p* = .82
BIM - 2^nd^ order Motor	19.75 (3.21)	23.64 (3.99)	−2.96 (29)	*p* = .006
BIM 2^nd^ order non-planning	19.81 (4.13)	22.67 (4.86)	−1.76 (29)	*p* = .088
BIM 2^nd^ order Total Score	56.56 (8.22)	63.43 (10.04)	−2.06 (29)	*p* = .049

The sample was split into two groups, high and low schizotypy traits, using predetermined cutoffs (Cohen, Callaway, Najolia, Larsen & Strauss, 2012; Dinzeo & Thayasivam, 2021) based on scores on the schizotypal personality questionnaire (SPQ, Raine, 1991). There were significant differences on the SPQ between low (*M*_low_ =41.44, *SD*_low_ = 9.74) and high (*M*_high_ = 69.20, *SD*_high_ = 10.84) schizotypy groups *t* (29) = −7.51, *p* < .001, *Hedges’ g* = 10.56.

#### 2.1.1. Exclusion criteria

Participants were excluded if they met any of the following: history of head injury leading to concussion or loss of consciousness within the past six months; diagnosis of a learning disorder or a neurological condition (e.g., epilepsy); current or regular medication use (apart from oral contraceptives); or received electroconvulsive therapy within the past year or a self-reported diagnosis of a severe mental illness (i.e., bipolar disorder or schizophrenia as per the DSM-5). Due to the known comorbidities of those with high schizotypy traits (Lewandowski et al., 2006), this study did not exclude participants if they had a self-reported diagnosis of depression or anxiety to ensure the study was more generalizable to the population being studied. Groups had an almost even split of individuals with a self-reported diagnosis of depression or anxiety between the two groups with five individuals with a diagnosis in the low schizotypy group and six in the high schizotypy group.

### 2.2. EEG recording

ERPs of interest were extracted from the EEG activity, which was recorded for each participant using an electrode cap with Ag^+^/Ag^+^-Cl^-^ ring electrodes at 32 scalp sites according to the 10–20 system of electrode placement, including 3 midline sites (frontal [F_z_], central [C_z_], and parietal [P_z_]), 3 left hemisphere (frontal [F_3_], central [C_3_], and temporal [T_7_]), and 3 right hemispheres (frontal [F_4_], central [C_4_], and temporal [T_8_]). Electrodes placed on the right and left mastoid, as well as mid-forehead, served as reference and ground channels, respectively. Electro-oculogram activity was recorded from supra-/sub-orbital and external canthi sites via bipolar channels. All electrode impedances were below 5 kΩ, and all electrical activity was recorded using BrainVision Recorder software with an amplifier bandpass of DC to 100 Hz and digitized at 500 Hz. Data were then stored on a hard drive for offline analysis using the BrainVision Analyzer (Brain Products GmbH, Munich DC) software.

#### 2.2.1. Neurophysiological task

The paired-stimulus paradigm was comprised of 32 paired clicks (S_1_–S_2_) with an inter-click interval of 500 ms and an interpair interval of 8 s (Zouridakis & Boutros, 1992). The paired click paradigm was presented binaurally through headphones with the 100 μs clicks presented with an intensity level of 80 dB (SPL) to participants while they observed a silent neutral movie. To analyze each component, data were segmented separately into the 1^st^ (S1) and 2^nd^ (S2) clicks. Both S1 and S2 were segmented from −50 to 120 ms relative to the onset of the click. For the P50 analysis, electrical epochs were then digitally filtered using low and high filters of 10–50 Hz, respectively, to increase the signal-to-noise ratio. The N100 and P200 were analyzed within epochs of 400 ms duration (including 50 ms pre-stimulus) using a frequency filter ranging from .1 to 30 Hz. All epochs were then ocular-corrected for eye movement (residual movement and blinks) using the Gratton and Coles algorithm (Gratton, Coles, & Donchin, 1983) and baseline-corrected (relative to the −50 ms pre-stimulus segment). Epochs containing voltages above 50 μV were excluded from the analysis, and the remaining data were used for the final ERP averaging.

Taken from the Cz scalp site, the site of maximal amplitude, P50 amplitudes were measured as the amplitude of the most positive peak from 40 to 80 ms relative to click onset. Peak picking was done using the averaged waveforms for each participant using a semi-automatic process with each peak verified by the first author AMF. To ensure accurate detection of the P50, additional constraints were applied to the analysis protocol (Nagamoto et al., 1991; Zouridakis & Boutros, 1992): the P50 had to be observed in at least one of the other central electrode sites (C_3_ and C_4_), and S_2_ P50 activity must peak within 10 ms of the peak observed from the P50 of S_1_. The N100 was defined as the largest negative deflection between 80 and 180 ms, while the P200 was defined as the largest positive deflection between 100 and 250 ms (Gooding, Gjini, Burroughs, & Boutros, 2013).

For each component, we calculated sensory gating in two ways following Broyd et al. (2013), and gating ratios (S_2_/S_1_) for each component (i.e., rP50, rN100, rP200: S_2_/S_1_) were generated, as well as difference measures (S_1_–S_2_) of sensory gating for each component (i.e., dP50, dN100, dP200), which have been used more recently (Broyd et al., 2013) to provide a more reliable index of sensory gating.

### 2.3. Questionnaires

#### 2.3.1. Schizotypal Personality Questionnaire – Brief Revised (SPQ-BR)

All participants completed the SPQ-BR to quantify their schizotypy traits at the time of testing. The SPQ-BR is a 32-item self-report scale that assesses schizotypy traits in healthy populations and is validated in a university sample (Cohen, Matthews, Najolia, & Brown 2010). Participants were asked to respond to each question on a five-point Likert scale, where 0 = “strongly disagree” and 4 = “strongly agree,” and higher scores indicate higher schizotypy traits. This overall scale can be further divided into four subscales: constricted affect (CA), social anxiety (SA), disorganized (DO), and ideas of reference (IR) based on a previously conducted factor analysis (Cohen et al., 2010).

#### 2.3.2. Sensation Seeking Scale Form V

All participants completed the Sensation Seeking Scale Form V (Zuckerman, Eysenck, & Eysenck, 1978) which is a 40-item measure comprised of four primary scales (disinhibition, boredom susceptibility, thrill and adventure-seeking, and experiences seeking). Each of the four subscales forced participants to choose between two options, assessing their desire for social and sexual disinhibition, aversion to daily routines and routine behaviors, their interest in participating in dangerous sports and activities, and their interest in travel and nonconforming lifestyles (Zuckerman et al., 1978). Scores were summed for each of the four subscales, and a total score was generated for each participant.

#### 2.3.3. Barratt Impulsivity Scale

The Barratt Impulsivity Scale (Patton et al., 1995) is a 30-item measure used to assess impulsive traits and behaviors. Participants indicated on a four-point Likert scale (ranging from 1 = never/rarely to 4 = almost always/always) how frequently they exhibit each impulsive or non-impulsive activity or behavior, (e.g., “I am a careful thinker”). The Barratt Impulsivity Scale has six first-order and three second-order factors that assess broad ranges of impulsivity such as self-control, attentional, and motor. Separate total scores were generated for each first-order and second-order factor, and an overall total score was calculated for each participant.

### 2.4. Study procedure

Upon arrival to the laboratory, participants completed informed procedures consent followed by a series of questionnaires on demographics, impulsivity, and personality characteristics. Testing took place between 11 am and 1 pm to control for time of day effects (Hines, 2004). Participants were asked to refrain from drug (e.g., tobacco, alcohol, cannabis) and medication use (apart from oral contraceptives) beginning midnight the night before their testing session. Verbal confirmation of abstinence was obtained upon arrival at the laboratory, and testing sessions were rescheduled if abstinence instructions were not followed.

Study procedures were conducted following clearance from the Mount Saint Vincent University Research Ethics Board (REB #1020019). The authors assert that all procedures contributing to this work comply with the ethical standards of the relevant national and institutional committees on human experimentation and with the Helsinki Declaration of 1975, as revised in 2008.

### 2.5. Statistical analysis

Some participants were excluded from data analysis due to uninterpretable data (e.g., too few artifact-free epochs). Specifically, data for two participants were removed, leaving the final data set of *N* = 31. Two groups were then generated using the SPQ-BR to create low and high schizotypy (STPY) groups. Groups were created by using predetermined cutoffs (Cohen et al., 2012; Dinzeo & Thayasivam, 2021) where scores falling 1.65 SD above the mean were considered high STPY, while scores falling below the mean were considered low STPY. Any scores that fell within the mid-range were collapsed and added to the high group as only two participants fell in this “intermediate range.” The final groups were *n* = 16 low STPY and *n* = 15 high STPY.

Repeated-measures analyses of variances (ANOVAs) were carried out for P50 amplitudes using the Statistical Package for the Social Sciences (SPSS 25; SPSS Inc., Chicago, IL), with one between-group factor (two levels: low STPY and high STPY) and one within-group factor (two levels: Stimulus one (*S*_1_) and Stimulus two (S_2_)). In addition to this analysis, amplitudes for each of the two stimuli were used to calculate gating ratios (rP50: S_2_/S_1_) and difference (dP50: S_1_–S_2_) scores for each participant. Independent samples *t*-tests were performed to assess group differences between low and high STPY groups and gating ratios (rP50) and difference scores (dP50). The same procedure was carried out for the other components of the MLAER (i.e., N100, P200).

Correlational analyses were conducted to measure relations between behavioral and demographic measures and the amplitude of our ERPs of interest (P50, N100, P200). Bivariate correlations (Spearman’s rho) using a two-tailed significance level were run to examine associations between demographic/clinical variables and ERP outcomes.

## 3. Results

All 31 participants (low STPY = 16 and high STPY = 15) were included in the analysis of the paired click paradigm. A Chi-square test for independence was performed to examine differences between low and high STPY groups on measures of gender, medication status, and diagnosis status. We found no significant differences between groups on gender, self-reported diagnosis, or medication status (*p*-values ranging from .716 to 1.0). Independent samples t-tests were performed to determine if there were any group differences on the four SPQ-BR subscales, the low STPY group had significantly lower scores on the overall SPQ-BR as well as all four subscales of the SPQ-BR, further supporting the fact that they did have a significant difference in schizotypy ratings compared to the high STPY group (see Table 1, *p*-values < .001). There were no significant differences between groups on the Sensation Seeking Scale or any of its submeasures; however, there were significant differences in scores on the Barrett Impulsivity Scale; specifically, the overall impulsivity score and motor impulsivity scores (both 1^st^ and 2^nd^ order) were higher for those in the high schizotypy group (see Table 1).

### 3.1. Amplitude

**Figure 1.**
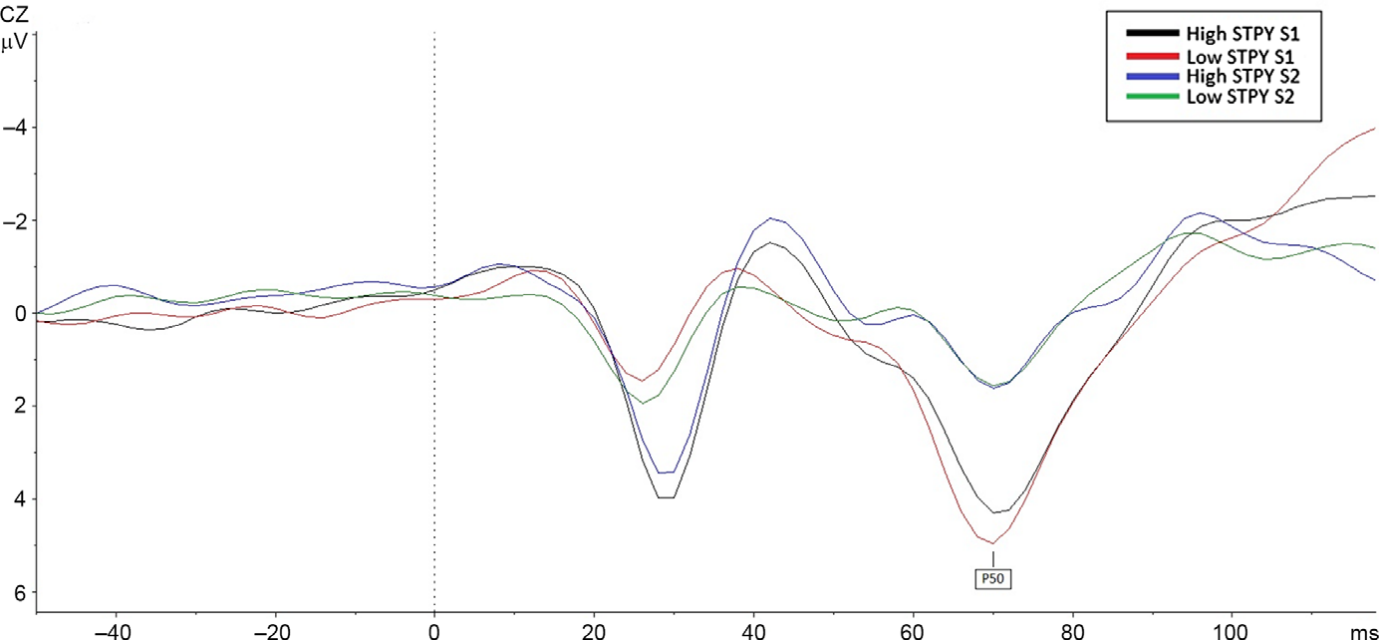
Comparison of P50 amplitudes between high and low STPY groups on the paired click paradigm. The figure shows the response to both S_1_ and S_2_.

A repeated-measures ANOVA, with Bonferroni adjustment for multiple comparisons, was performed to assess group differences on P50 amplitudes. There was a main effect of stimuli *F* (1,28) = 56.54, *p* < .001, such that amplitudes for S_1_ (*M* = 3.91, *SE =* .41, *95% CI* [3.07–4.75]) were larger than amplitudes for S_2_ (*M* = 1.15, *SE =* .25, *95% CI* [.064–1.65]), and this was consistent across groups (*p* < .001, for both high and low STPY). There were no group differences (*p* = .85) and no group by stimulus (P50) interactions (*p* = .54, see Figure 1).

Ratio and difference scores showed no main effect of group, suggesting no P50 gating differences between high and low STPY conditions (*p* = .89, *p* = .98, respectively). Effect sizes and confidence intervals corroborate this finding (rP50: *g* = .07, *95% CI* [.054–.47], dP50: *g* = .006, *95% CI* [−1.17 to 1.20]).

**Figure 2.**
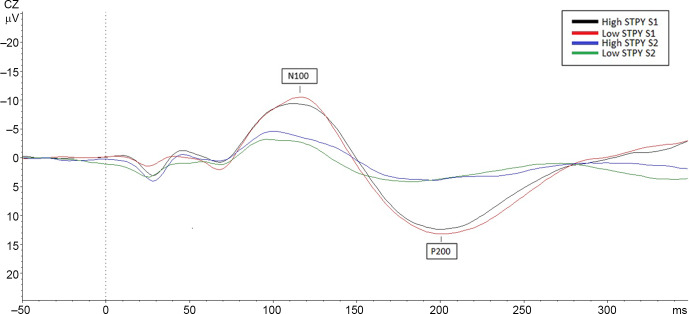
Comparison of N100 and P200 amplitudes in the high and low STPY groups, the figure shows the response to both S_1_ and S_2_ during the paired click paradigm.

There were no main or interaction effects for the N100 amplitude for the paired click paradigm (*p*-values ranging from .46 to .55, see Figure 2), confidence intervals, and effect sizes substantiate this finding (Stimulus: *g* = .11, *95% CI* [−1.12 to .61], group: *g* = .19, *95% CI* [−1.02 to 1.91]). Ratio and difference scores showed no significant group differences and no group by stimulus interactions (*p*-values ranging from .46 to .70) confidence intervals and effect sizes verify this finding (see Table 2).

**Table 2.** Repeated measures ANOVA findings separated by ERP of interest.

		df	F-value	Significance (*p* -value)	Effect Size *(g*-value)	Confidence Intervals (95% [low, high])
		Amplitude				
P50						
	Group	1,28	.034	*p* = .86	*g* = .15	[−1.06–1.27]
	Stimulus	1,28	56.54	*p* < .001	*g* = 1.52	[2.01−3.51]
	Group*Stimulus	1,28	33.12	Low*: p* < .001 High: *p* < .001	Low*: g* = 1.62 High: *g* = 1.41	Low: [1.92−4.05] High: [1.47–3.59]
	Stimulus*Group	1,28	.16	S_1_: *p* = .69 S_2_: *p* = .81	S_1_: *g* =.12 S_2_: *g* =.089	S1: [−1.35–2.01] S2: [−1.13−.88]
N100						
	Group	1,28	.39	*p* = .54	*g* = .20	[−1.02–1.91]
	Stimulus	1,28	.36	*p* = .55	*g* = .11	[−1.12−.61]
	Group*Stimulus	1,28	.92	*p* = .35	Low: *g* = .84 High: *g* = .44	[11.72–17.95] [2.50–6.77]
	Stimulus*Group	1,28	.88	*p* = .36	S_1_: *g* = 1.34 S_2_: *g* = .22	[−.90–2.42] [−1.62–1.88]
P200						
	Group	1,28	.067	*p* = .80	*g* = .36	[−2. 87–3.70]
	Stimulus	1,28	106.06	*p* < .001	*g* = 3.87	[7.47–11.18]
	Group*Stimulus	1,28	.92	*p* = .35	Low: *g* = .84 High: *g* = .44	[11.72–17.95] [2.50–6.77]
	Stimulus*Group	1,28	.36	*p* = .56	S_1_: *g* = .84 S_2_: *g* = .43	[−3.12–5.69] [−3.46–2.56]

Repeated-measures ANOVA revealed a main effect of the stimulus on P200 amplitudes for the paired click paradigm *F* (1,28) = 106.06, *p* < .001, such that amplitudes for S_1_ (*M* =14.19, *SD =* 5.83) were larger than S_2_ (*M* = 4.86, *SD* = 3.97, *g* = 2.0, *95% CI* [7.47–11.18]). There were no main effects of group (*p* = .80; see Figure 2) or a group by stimulus interaction (*p* = .35). rP200 and dP200 showed no difference between groups (*p*-values ranging from .35 to .22).

### 3.2. Correlations

Spearman’s rho bivariate correlations were performed to measure the relationship between the three ERPs of interest and the demographic and psychological variables. rP50 positively correlated with gender (*r* = .43, *p* = .018), suggesting that female participants may have larger p50 ratios and thus worse gating. rP50 also positively correlated with diagnosis status (i.e., self-reported diagnosis of depression or anxiety), such that those who had a diagnosis of a mental illness were more likely to have higher rP50 scores (*r* = .37, *p* = .047) and thus worse gating. dP50 showed a negative correlation with gender (*r* = −.37, *p* = .043) such that female participants were more likely to have smaller differences and thus again worse gating. Finally, the P50 S_1_ and S_2_ amplitude correlated with Barrett Impulsivity, cognitive complexity scale (S_1_: *r* = .38, *p* = .037; S_2_: *r* = .39, *p* = .006).

The P50 correlation with diagnosis status were followed up with an independent samples *t*-test to determine if there were differences between those who had a diagnosis of mental illness (i.e., self-reported diagnosis of depression or anxiety) and those that did not on rP50 and dP50 sensory gating. We found that those who had a diagnosis of depression or anxiety (*M* = .48, *SD* = .41) had larger gating ratios than those without a diagnosis (*M* = .15, *SD* = .32, *p* = .02, *g* = .93, *95% CI* [−.63 to (−.030)]), and this same trend was shown in the dP50 data whereby those with a diagnosis (*M* = 1.81, *SD* = 1.59) had smaller difference scores compared to those without a diagnosis (*M* = 2.96, *SD* = 1.42, *p* = .051, *g* = .77, *95% CI* [−.0048, 2.29]), thus suggesting those with a diagnosis of mental health or psychiatric conditions performed worse on measures of sensory gating. To determine if groups with a diagnosis were different from those without a diagnosis on SPQ measures, we ran another independent samples *t*-test, and no significant differences were found (*p*-values range from .56 to .86). Finally, to better understand the relationship between rP50, dP50, and gender, a final independent samples *t*-test was performed. We found no significant differences between male and female participants on rP50 and dP50 measures of sensory gating (*p*-vales ranging from .43 to .99).

For the N100, we found a significant correlation between N100 S_1_ amplitude (*r* = −.40, *p* = .03) with the following subscales from the Barrett Impulsivity Scale – perseverance index (*r* = −.40, *p* = .03), cognitive complexity (*r* = −.44, *p* = .016), 2^nd^ order non-planning (*r* = −.43, *p* = .019), and Barrett Impulsivity total score (*r* = −.42, *p* = .024) suggesting that when these scores increase, N100 S_1_ amplitude decreases (i.e., becoming more positive). N100 S_2_ amplitude was also correlated with cognitive complexity (*r* = −.58, *p* < .001). rN100 was found to positively correlate with diagnosis status (i.e., self-reported diagnosis of depression or anxiety; *r* = .36, *p* = .048), suggesting that larger ratios (worse gating) were associated with having a self-reported diagnosis of a mental illness.

rP200 correlated with the interpersonal traits subscale of the SPQ (*r* = .42, *p* = .021) such that higher interpersonal traits were associated with larger ratios (i.e., worse gating). Additionally, the experience-seeking subscale of the sensation-seeking scale was positively correlated with rP200 (*r* = .45, *p* = .012) and dP200 scores (*r* = −.48, *p* = .008), suggesting higher scores on this subscale are related to worse sensory gating ability (i.e., higher ratios and lower difference scores). This inventory also correlated with P200 S_2_ amplitude (*r* = .38, *p* = .039), suggesting higher scores on the experiences inventory were related to larger S_2_ amplitudes. Finally, dP200 negatively correlated with the social anxiety measure of the SPQ (*r* = −.39, *p* = .032), suggesting that worse social anxiety would lead to smaller P200 difference scores (i.e., worse sensory gating).

## 4. Discussion

The aim of this study was to better understand the relation of schizotypy traits with sensory gating ability in a sample of healthy individuals. Sensory gating was assessed through the paired click paradigm and mid-latency evoked responses (i.e., P50, N100, and P200), while schizotypy traits were assessed through the SPQ-BR which was used to classify participants into “high” and “low” schizotypy groups. Based on prior work (Clementz et al., 1998; Croft et al., 2001, 2004; Hazlett et al., 2015; Myles-Worsley et al., 2004; Olincy et al., 2010; Wan et al., 2006, 2007), we hypothesized that those with the highest schizotypy scores would have the greatest reductions in P50-indexed sensory gating.

While we found the anticipated main effects of stimulus for the P50 and P200 ERPs (i.e., S_1_ amplitude > P50 S_2_ amplitude), we found no group differences on any of our measures of sensory gating. Some interesting correlations emerging from our data suggest possible associations between schizotypy traits and our sensory gating variables of interest (i.e., P50, N100, and P200). Additional correlations were also found between these sensory gating measures and measures of impulsivity and between the sensory gating measures and indexes of sensation-seeking behaviors. These correlations, however, were not all in the expected direction. We found that individuals who scored high on the social anxiety measure of the SPQ-BR were more likely to have worse attention allocation (indexed by the dP200), suggesting that participants who had high levels of social anxiety had worse downstream sensory gating ability. Additionally, in agreement with prior work (Houston & Stanford, 2001; Lawson et al., 2012; Lijffijt et al., 2012; Zheng et al., 2010), we found a significant correlation suggesting that individuals high on sensation-seeking (specifically the experience-seeking subscale of the SSS) were more likely to have worse attention allocation, as indexed by the rP200. While these correlations relate to schizotypy, they suggest that the deficits shown by our sample may come from attention deficits as opposed to early sensory processing ability and cognitive deficits related to high schizotypy traits. This finding further provides evidence that our sample was not displaying the sensory gating deficit we would have expected to see in those with high levels of trait schizotypy.

Correlational analyses suggested that participants with a self-reported diagnosis of a mental or psychiatric illness (excluding schizophrenia and bipolar disorder) would have worse P50 sensory gating ability compared to those without a diagnosis. Follow-up analyses showed that there was a significant difference in the sensory gating ability of those with and without a diagnosis, suggesting that those with a diagnosis had worse gating; however, these groups did not differ on SPQ-BR scores, suggesting they did not differ on any schizotypy traits. Given that we had a diverse sample with several different disorders present, it was not possible to parse out which disorder(s) were driving this relationship.

Consistent with prior work (Hetrick et al., 1996; Lijffijt et al., 2009; Patterson et al., 2008), we found a correlation suggesting that females had worse sensory gating ability compared to males; however, there were no significant differences between males and females on any of the sensory gating measures, and there were no significant differences between groups on gender. This null finding is possibly due to our relatively small cell sizes (*n* < 10/per cell).

While our findings suggest that healthy controls with schizotypy traits (high vs low) do not differ in their sensory gating ability, our sample had relatively low levels of schizotypy given the maximum score is 128 on the SPQ-BR and our sample had a range of 23–88. Thus, even those with the higher SPQ-BR scores were not in the highest possible range. It is possible that these participants, while they had schizotypy traits, were not high enough on the SPQ-BR scale to experience the sensory gating deficits that have previously been reported in the literature (Clementz et al., 1998; McGhie & Chapman, 1961; Olincy et al., 2010). It is of value to note that (Clementz et al., 1998; McGhie & Chapman, 1961; Olincy et al., 2010) did not use the SPQ as a measure in their studies, and therefore a direct comparison between studies cannot be made. One possible explanation for this difference is the education status of our participants. Despite the SPQ-BR being validated in a university sample (Cohen et al., 2010), our participants were gathered from a predominantly university student sample suggesting that the level of severe cognitive dysfunction we would expect with a high schizotypy group (Evans et al., 2007; Wan et al., 2006) was relatively minimal.

### 4.1. Limitations

A notable limitation of the study was the relatively low schizotypy scores in the “high” cutoff group. This feature could be partially due to the fact that most of our participants were recruited from a university sample and therefore limits the generalizability of our findings. Additionally, we had a relatively small sample size, and our sample did not have equal representations of each sex; therefore, future studies should ensure a larger sample and an equal distribution so that the interaction between schizotypy and biological sex can be examined. Our sample did not exclude or ask about genetic vulnerability to schizophrenia, and it is possible that our participants were at high risk or had a first-degree relative that had schizophrenia or bipolar disorder, which could impact the findings; however, these data were not collected. For future studies, we plan to collect this information to better understand the sample. We also suggest that future studies aim to better differentiate the relationship between sensory gating and the refractory period for the N100 ERP. Based on prior work (Budd et al., 1998), it has been suggested that the decrease in N100 amplitude is reflective of a refractory period that goes away with longer ISI; however, the current paradigm does not allow for us to test the differences between this potential refractory period and the sensory gating processes. Future studies should aim to better disentangle this relationship.

Finally, some participants in our sample had a self-reported diagnosis of other mental illnesses at the time of testing (i.e., depression, anxiety), while this is a limitation and may cloud the findings, and it does make our findings more generalizable to the general population with high schizotypal traits. In the future, however, more information regarding these diagnoses would be beneficial, including questionnaires verifying the mental illness and specific diagnoses.

## 5. Conclusions

While this study does not show differences between relatively low and high schizotypy groups, it does suggest that some of our participants may have been experiencing deficits in attention allocation, a downstream cognitive processing measure relative to individual differences in interpersonal traites related to schizotypy. Scores on the SPQ-BR suggest that our sample was not close to the high end of the schizotypy traits which may help explain why no differences were found. This null finding may be explained partially by the sample which consisted of educated participants in a bachelor’s degree program, suggesting that they were not suffering from severe cognitive deficits that would impede their cognitive performance. This study helps to show that individuals with lower- and mid-range levels of schizotypy do not suffer from the same cognitive deficits in sensory gating and attention filtering/allocation that are seen in those with high levels of schizotypy but may have minor deficits in the later stream attention allocation processing depending on their levels of sensation-seeking, inhibition, and social anxiety. This research shows the importance of including all levels of schizotypy ratings in clinical research as we can gain a clearer view of the impact of schizotypy on the brain and cognitive functioning in those with “high” levels of schizotypy. Additionally, this work highlights the importance of including measures of important factors such as impulsivity and sensation-seeking to better understand what aspect of schizotypy may be driving these sensory gating deficits reported in the literature (Clementz et al., 1998; Croft et al., 2001, 2004; Hazlett et al., 2015; Myles-Worsley et al., 2004; Olincy et al., 2010; Wan et al., 2006, 2007).
